# Pulse Oximetry Screening for Critical Congenital Heart Disease: A Four-Year Experience in Qatar

**DOI:** 10.7759/cureus.105810

**Published:** 2026-03-25

**Authors:** Rawia Abu Jarir, Nuha Nimeri, Azzam Alsaid, Reema Kamal, Linda Ibrahim, Faten Haridy

**Affiliations:** 1 Neonatal Intensive Care Unit (NICU), Women’s Wellness and Research Center (WWRC), Hamad Medical Corporation, Doha, QAT; 2 Cardiology, Hamad Medical Corporation, Doha, QAT; 3 Critical Care, Women’s Wellness and Research Center (WWRC), Hamad Medical Corporation, Doha, QAT

**Keywords:** congenital cardiac anomalies, critical congenital heart disease screening threshold, neonatal intensive care unit (nicu), pulse oximetry, screening program

## Abstract

Background: Critical congenital heart disease (CCHD) is a major cause of neonatal morbidity and mortality. Early postnatal detection using pulse oximetry screening (POCC) facilitates timely diagnosis and intervention.

Objective: To evaluate the performance of POCC for detecting CCHD among newborns delivered at the Women’s Wellness and Research Centre, Qatar, between 2017 and 2020.

Methods: A retrospective chart review of all live births between January 2017 and December 2020 was conducted. Data from screening registries and patient records were analysed using descriptive and comparative methods.

Results: Of 68,150 live births, 141 CCHD cases were identified: 74 (52%) antenatally, 33 (23%) clinically, and 34 (24%) via POCC. Among POCC cases, eight were true positives and 26 false positives (positive predictive value 23.5%). Pulmonary hypertension of the newborn (PPHN) was the most commonly identified lesion.

Conclusion: POCC complements antenatal and clinical diagnosis, identifying silent but critical cases. Universal screening should remain standard practice to ensure early detection and improved outcomes.

## Introduction

Congenital heart disease (CHD) refers to any structural or functional abnormality of the heart’s circulation that is present at birth. It is the most common serious congenital disorder, affecting nearly 1% of all live births [1]. CHD represents a leading cause of infant mortality due to congenital disabilities, accounting for a significant proportion of neonatal and infant deaths attributed to congenital anomalies [2]. Unfortunately, the causes of most CHD cases remain largely unknown, which means that effective primary prevention strategies are elusive [3].

Within the broad spectrum of CHD, a subset is classified as critical congenital heart disease (CCHD). Approximately one in every 100 newborns has some form of heart defect, and about one-quarter of these cases are deemed critical [1]. Critical CHDs are severe cardiac malformations that typically require surgical or catheter-based intervention within the first year of life to ensure survival [1]. Many infants with CCHD may appear healthy at birth and during the first hours or days of life [4]. If undetected, these babies can deteriorate rapidly, developing hypoxemia, cardiovascular collapse, organ failure, or even death within days [4,5].

Prenatal screening with ultrasound can identify some major anomalies, but sensitivity is limited. Even in developed healthcare systems, routine antenatal ultrasound detects only about 50% of serious CHD cases requiring surgery in the first year [6]. Detection rates vary by lesion and expertise, with many infants born in low-risk settings where CHD is unsuspected [6,7]. One U.S. study estimated that roughly 30% of infants with CCHD are not diagnosed until after the first three days of life [5], and in England, about 25% were detected only after discharge [6].

To address this gap, newborn pulse oximetry screening (POCC) has been introduced in many countries. Pulse oximetry is a simple, non-invasive test that measures oxygen saturation and can flag hypoxemia before clinical deterioration. Because of its utility, the American Academy of Paediatrics and public health authorities recommend universal CCHD screening before discharge [8,9]. In the United States, CCHD screening was added to the Recommended Uniform Newborn Screening Panel in 2011, and by 2018, all states had adopted it [8,9]. The test is quick, inexpensive, and minimally disruptive, and studies confirm it is safe, highly specific, and cost-effective [5,10].

Early detection through pulse oximetry enables timely confirmatory echocardiography and initiation of interventions (e.g., prostaglandin infusion, surgical referral) before the infant collapses [9,11]. Evidence shows that states implementing mandatory screening saw a 33% reduction in early infant deaths from CCHD compared with states without screening [9]. Thus, routine POCC represents a reliable, safe, and cost-effective public health strategy to improve neonatal outcomes and reduce mortality from CCHD [4,9-11].

CCHD remains a leading cause of neonatal morbidity and mortality worldwide. Early postnatal detection through POCC improves timely diagnosis, facilitates early intervention, and reduces preventable deterioration. Despite widespread global adoption, variability in screening implementation, compliance, detection rates, and follow-up pathways persists across institutions. Data on the real-world performance of POCC within individual healthcare systems remains essential for ensuring quality, safety, and effectiveness. Continuous local audit enables identification of gaps in practice, false-negative and false-positive rates, and the overall impact of screening on neonatal outcomes.

Although POCC has been widely adopted internationally, limited published data exist regarding its implementation and diagnostic yield in Qatar. Evaluating institutional screening performance provides important evidence to guide quality improvement and support national neonatal screening strategies.

Therefore, we conducted this study to review our institutional experience and to evaluate the performance of POCC screening in our unit. Our objective was to assess screening performance, diagnostic yield, and clinical outcomes in order to inform quality improvement and optimise neonatal cardiovascular care and support earlier detection and timely clinical management.

Screening babies for pulse oximetry before discharge home has many advantages. It can ensure patient safety by diagnosing CCHD before an infant becomes symptomatic, which is cost-effective, and by developing strategies for implementing safe and efficient CCHD screening across all neonatal intensive care units (NICUs) in Qatar.

## Materials and methods

This retrospective study was conducted at the Women’s Wellness and Research Centre (WWRC) in Doha, Qatar, to evaluate universal newborn screening for CCHD from January 2017 to December 2020. All live-born infants admitted to the postnatal wards during the study period were included in the screening cohort.

POCC was performed for all newborns prior to discharge, typically at approximately 24 hours of life. In some instances, screening was conducted earlier due to bed-capacity constraints. Data were obtained from the newborn screening registry and supplemented by medical records and Cerner electronic health record entries.

All cases of CHD identified during the study period were reviewed. Detailed analysis was performed on infants diagnosed with CHD through antenatal screening, clinical examination, or POCC.

The screening protocol followed institutional guidelines adapted from the American Academy of Paediatrics (AAP) recommendations, based on a modified CCHD screening algorithm. Oxygen saturation measurements were obtained from both pre-ductal (right hand) and post-ductal (either foot) sites. A screen was considered abnormal if any of the following criteria were met: (1) oxygen saturation <90% in either extremity; (2) oxygen saturation of 90-94% in both extremities on repeated measurements; or (3) a ≥3% difference between pre- and post-ductal saturations. Infants with borderline results underwent repeat screening according to institutional protocol, and those with persistent abnormal findings were referred for echocardiographic evaluation and further clinical assessment.

Due to the retrospective design and reliance on in-hospital data, complete identification of false-negative cases after discharge was not feasible. Therefore, the sensitivity and specificity of the screening test were not calculated.

Statistical analysis

Data were analysed using descriptive statistics, including frequencies, percentages, median values, and ranges. With approximately 15,000 annual deliveries and a CHD incidence of 8 per 1,000, an estimated 120 cases per year were anticipated. To ensure comprehensive coverage, all diagnosed cases of CHD during the study period were included.

## Results

During the study period, 68,150 live births were recorded, among which 141 cases of CCHD were identified. Of these, 74 cases (52.5%) were diagnosed antenatally, 33 cases (23.4%) were detected clinically postnatally, and 34 cases (24.1%) were identified through POCC prior to discharge.

In our cohort of 68,150 live births, POCC screening identified eight previously undiagnosed cases of CCHD, corresponding to a detection yield of approximately 1.17 per 10,000 live births.

**Figure 1:**
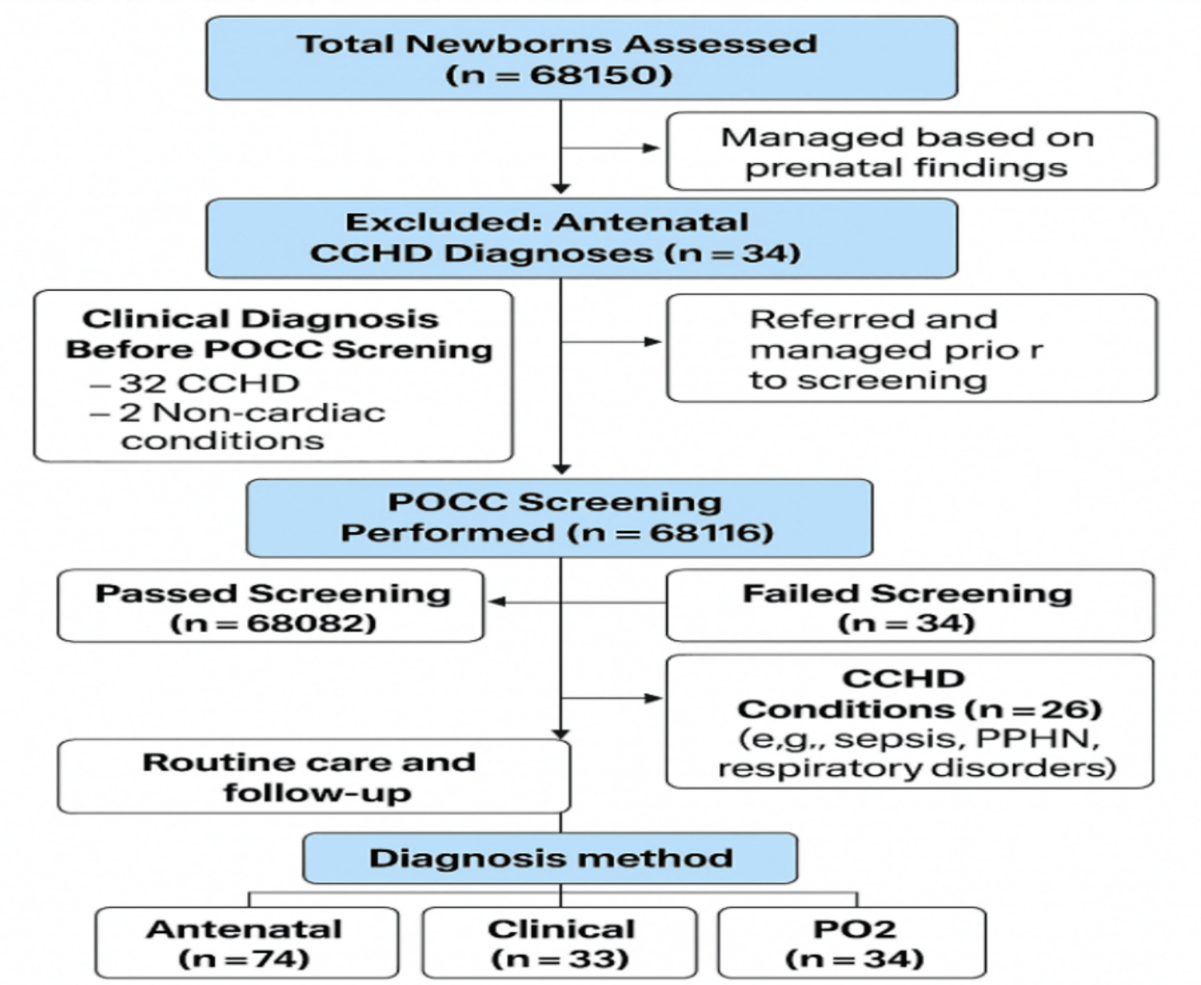
Study algorithm CCHD: critical congenital heart disease; POCC: pulse oximetry screening; PPHN: persistent pulmonary hypertension of the newborn; PO2: pulse oximetry

Among infants screened with POCC, eight were confirmed to have CCHD, while 26 had non-cardiac diagnoses, yielding a positive predictive value of 23.5%. The most frequent non-cardiac condition detected through POCC was persistent pulmonary hypertension of the newborn (PPHN). Infants identified via POCC generally exhibited fewer classical clinical signs, such as murmurs or cyanosis, but had a higher incidence of absent or weak femoral pulses, consistent with duct-dependent lesions (Figure 1).

Table 1 summarises the demographic and clinical characteristics of newborns assessed for suspected cardiac disease. The majority were term infants (median gestational age, 39 weeks) and were assessed at a median age of two hours. Sex distribution was balanced, and 78.1% of infants were non-Qatari. A heart murmur was present in 77.2% cases, with a near-equal distribution between low- and high-intensity murmurs. Tachypnea was observed in 54.4% of infants, and cyanosis in 29.2%. Femoral pulses were palpable in 94.7%, indicating that this important sign was absent in only a small proportion of cases.

**Table 1:** Demographic and clinical characteristics of CCHD cases (n=141) GA: gestational age; CCHD: critical congenital heart disease; IQR: interquartile range; PO2: pulse oximetry

Characteristics	Diagnosis method						Total (n=141)	
	Antenatal (n=74)		Clinical (n=33)		PO2 (n=34)			
	n	%	n	%	n	%	n	%
GA (median, IQR)	39 (37-39)		39 (36-39)		39 (38-40)		39 (37-39)	
Age in hours (median, IQR)	1 (1-2)		24 (6-40)		12 (12-24)		6 (1-19)	
Sex								
Male	34	45.9	17	51.5	17	50.0	68	48.2
Female	40	54.1	16	48.5	17	50.0	73	51.8
Nationality								
Qatari	10	13.5	13	39.4	8	23.5	31	22.0
Non-Qatari	64	86.5	20	60.6	26	76.5	110	78.0
CCHD anomaly								
No	0	0.0	1	3.0	26	76.5	27	19.1
Yes	74	100.0	32	97.0	8	23.5	114	80.9
Heart murmur								
No	16	21.6	8	24.2	29	85.3	53	37.6
Yes	58	78.4	25	75.8	5	14.7	88	62.4
Intensity								
Low	36	59	13	48.1	1	20.0	50	58.7
High	25	41.0	14	51.9	4	80.0	43	46.2
Cyanosis								
No	53	72.6	23	69.7	22	88.0	98	74.8
Yes	20	27.4	10	30.3	3	12.0	33	25.2
Tachypnea								
No	35	47.3	13	39.4	31	91.2	79	56.0
Yes	39	52.7	20	60.6	3	8.8	62	44.0
Femoral pulse								
No	2	2.7	3	9.1	14	42.4	19	13.6
Yes	72	97.3	30	90.9	19	57.6	121	86.4

These findings highlight that, although clinical signs remain important for early suspicion, a substantial proportion of infants with CCHD present without overt features, underscoring the importance of systematic screening.

As detailed in Table 2, antenatal screening identified the largest proportion of CCHD cases (52.5%), most commonly tetralogy of Fallot (TOF; n=16) and dextro transposition of the great arteries (d-TGA; n=9). Clinical postnatal assessment identified 33 cases (23.4%), including TOF (n=8) and coarctation of the aorta (COA; n=5). POCC screening detected 34 cases (24.1%), with PPHN accounting for the majority (n=28) of POCC-positive diagnoses. Overall, the most frequent anomalies across all detection methods were TOF (n=24), PPHN (n=29), and d-TGA (n=15).

**Table 2:** Distribution of CCHD anomalies by diagnosis method (antenatal, clinical, and PO2) and total cases (n=141) COA: coarctation of the aorta; PO2: pulse oximetry; DORV: double outlet right ventricle; d-TGA: dextro transposition of the great arteries; HLHS: hypoplastic left heart syndrome; PPHN: persistent pulmonary hypertension of the newborn; TAPVR: total anomalous pulmonary venous return; TOF: tetralogy of Fallot; CCHD: critical congenital heart disease

Anomaly	Antenatal	Clinical	PO2	Total
Atrioventricular septal defect (AV canal defect)	12	1	0	13
COA	5	5	0	10
DORV	3	-	0	3
d-TGA	9	5	1	15
Ebstein anomaly	1	-	1	2
HLHS	10	3	1	14
PPHN	0	1	28	29
Pulmonary stenosis	1	3	0	4
TAPVR	1	4	1	6
TOF	16	8	0	24
Non-CCHD	12	3	2	21
Grand total	74	33	34	141

Of the 107 infants diagnosed before POCC screening (74 antenatal, 33 clinical), detection occurred early, facilitating timely referral and management. Among the remaining 68,116 infants screened, 34 failed POCC, of whom eight were confirmed to have CCHD. Although the false-positive rate was notable, all false-positive cases were associated with clinically significant non-cardiac conditions, supporting the role of POCC as a broader safety tool for detecting neonatal illness.

Table 3 demonstrates that antenatal and clinical diagnoses were associated with more symptomatic presentations, including higher rates of murmurs and tachypnea, whereas POCC-detected cases were predominantly asymptomatic, yet more likely to exhibit absent femoral pulses. Across all groups, sex distribution was balanced, and non-Qatari infants constituted the majority. Earlier diagnosis, particularly antenatal, was associated with earlier age at identification, potentially allowing improved clinical stabilisation and readiness for intervention.

**Table 3:** Comparison of clinical characteristics and diagnostic yield of antenatal, clinical, and PO2 screening methods for neonatal congenital heart disease GA: gestational age; CCHD: critical congenital heart disease; PO2: pulse oximetry

Characteristics	Diagnosis method						Total (n=141)	
	Antenatal (n=74)		Clinical (n=33)		PO2 (n=34)			
Variables	n	%	n	%	n	%	n	%
GA (median, IQR)	39 (37-39)		39 (36-39)		39 (38-40)		39 (37-39)	
Age in hours (median, IQR)	1 (1-2)		24 (6-40)		12 (12-24)		6 (1-19)	
Sex								
Male	34	45.9	17	51.5	17	50.0	68	48.2
Female	40	54.1	16	48.5	17	50.0	73	51.8
Nationality								
Qatari	10	13.5	13	39.4	8	23.5	31	22.0
Non-Qatari	64	86.5	20	60.6	26	76.5	110	78.0
CCHD anomaly								
No	0	0.0	1	3.0	26	76.5	27	19.1
Yes	74	100.0	32	97.0	8	23.5	114	80.9
Heart murmur								
No	16	21.6	8	24.2	29	85.3	53	37.6
Yes	58	78.4	25	75.8	5	14.7	88	62.4
Intensity								
Low	36	59	13	48.1	1	20.0	50	58.7
High	25	41.0	14	51.9	4	80.0	43	46.2
Cyanosis								
No	53	72.6	23	69.7	22	88.0	98	74.8
Yes	20	27.4	10	30.3	3	12.0	33	25.2
Tachypnoea								
No	35	47.3	13	39.4	31	91.2	79	56.0
Yes	39	52.7	20	60.6	3	8.8	62	44.0
Femoral pulse								
No	2	2.7	3	9.1	14	42.4	19	13.6
Yes	72	97.3	30	90.9	19	57.6	121	86.4

## Discussion

POCC for CCHD is now a standard component of newborn screening in the United States and many other countries, following its inclusion on the Recommended Uniform Screening Panel and endorsement by major societies, including the American Academy of Pediatrics and the American Heart Association. The rationale is that many forms of CCHD result in hypoxemia due to abnormal mixing of oxygenated and deoxygenated blood, which may not be clinically apparent, especially in infants with darker skin pigmentation, where cyanosis is less easily detected [12].

Diagnostic accuracy is well characterized in meta-analyses, and large cohort studies demonstrate that pulse oximetry is highly specific (99.9%) and moderately sensitive (76.3%) for CCHD, with a very low false-positive rate (0.14%) when performed after 24 hours of life. Sensitivity is lower for certain lesions, particularly those that do not cause hypoxemia in the immediate newborn period, such as COA and some left-sided obstructive lesions. For example, sensitivity for COA may be as low as 21%. Timing of screening is important; performing pulse oximetry after 24 hours reduces false positives compared to earlier testing [1].

This four-year retrospective study demonstrates the complementary roles of antenatal screening, postnatal clinical examination, and POCC in achieving early detection of CCHD. In our cohort, 52.5% of CCHD cases were identified antenatally and confirmed shortly after birth (median age one hour), reflecting a structured fetal anomaly pathway supported by specialist fetal cardiology services. However, nearly half of critical lesions remained undetected prenatally, reinforcing the limitations of antenatal ultrasound alone and the necessity for robust postnatal screening.

Among infants without antenatal diagnosis, 23.4% were identified clinically, and a further 24.1% through POCC before discharge, resulting in near-complete detection before hospital discharge. Although many affected neonates exhibited warning signs such as murmurs (62%) or tachypnea (44%), a substantial proportion lacked classical features: approximately one-third had no cyanosis, and over one-third had no murmur. This finding is consistent with evidence that routine newborn physical examination alone detects only a minority of CCHD cases, with detection rates as low as 31% reported in UK and European cohorts [12,13].

Absent or weak femoral pulses were uncommon overall (13.6%) but clustered among infants detected by POCC, where 42% had diminished pulses, suggesting duct-dependent systemic lesions. COA remains a diagnostic blind spot, with antenatal detection rates reported as low as 22% even in developed healthcare systems [13]. In our study, only half of the coarctation cases were detected antenatally, highlighting the added diagnostic value of POCC when combined with pulse assessment.

Our prenatal detection rate of approximately 53% aligns with international benchmarks. Global studies report antenatal detection rates ranging from 13% to 87%, with a median around 50% [14]. The UK National Congenital Heart Disease Audit similarly reports antenatal detection in approximately 54% of major CHD cases [15]. In contrast, highly specialized centers using extended cardiac views report detection rates exceeding 90% [16].

Detection in our cohort varied by lesion type. All atrioventricular canal defects and 71% of hypoplastic left heart syndrome (HLHS) cases were identified antenatally, whereas only 60% of transposition of the great arteries and 50% of coarctation cases were detected before birth. This mirrors international experience and underscores the vulnerability of outflow-tract and duct-dependent lesions to antenatal under-detection [14,16].

POCC identified 34 newborns with abnormal results, including eight true CCHD cases with no prior diagnosis. These included lesions such as d-TGA, HLHS, and total anomalous pulmonary venous return (TAPVR). These infants were clinically well at initial examination and would likely have been discharged undiagnosed without POCC. The addition of pulse oximetry increased overall detection to virtually 100% before discharge, consistent with population studies showing detection rates of 75-90% when POCC is combined with antenatal screening and clinical examination, compared with 50-60% without POCC [12,17-19].

A Jordanian prospective study similarly reported PPHN in 42% of screen-positive cases [20]. Evidence from a large UK regional neonatal unit over five years further supports these findings. Singh and Chen reported that pulse oximetry achieved 85.7% sensitivity and specificity exceeding 99%, identifying most undiagnosed critical lesions before discharge [21]. However, they also demonstrated that two-thirds of major CHDs requiring surgery within infancy may still be missed, emphasising that POCC serves as a safety net rather than a standalone solution [21].

In our cohort, most POCC-positive infants without CCHD had clinically significant non-cardiac conditions, most commonly PPHN, which accounted for 28 screen-positive cases. This is consistent with international data showing that over 80% of positive pulse oximetry results may be attributable to significant non-cardiac pathology such as sepsis, pneumonia, or respiratory distress [21]. These findings reinforce that so-called “false positives” often represent clinically meaningful early detections that enhance neonatal safety rather than generate unnecessary investigations [12,19].

The median age of POCC screening in our unit was approximately 12 hours of life. Early screening increases false-positive rates due to transitional circulation, with rates around 2.3% before 24 hours compared with 0.8% thereafter. The UK cohort similarly reported higher false-positive rates with early screening but highlighted the benefit of detecting time-sensitive non-cardiac pathology [21]. Importantly, in our study, no infant with true CCHD passed screening.

Overall, our findings demonstrate that a layered screening strategy combining antenatal ultrasound, vigilant clinical examination, and universal pulse oximetry achieves near-complete detection of CCHD before discharge. This approach aligns with international best practice and reduces the risk of post-discharge cardiovascular collapse.

However, consistent with international data, a proportion of serious and acyanotic CHDs remain undetected even with POCC [21]. Emerging adjuncts such as perfusion index measurement may enhance the detection of left-sided obstructive lesions and warrant further evaluation [20].

Although limited by its retrospective design and lack of formal statistical comparisons, the concordance between our findings and large international cohorts supports the validity of our conclusions. Universal POCC screening should remain a core component of neonatal safety, complementing antenatal and clinical assessment.

Our findings also align with broader epidemiological insights linking maternal obesity, diabetes, smoking, and environmental exposures to increased CHD risk, while advanced paternal age and consanguinity contribute through genetic mechanisms [19-21]. Qatar’s diverse population underscores the importance of integrating screening with preventive strategies such as preconception counseling and genetic risk education.

This study has several limitations. First, the retrospective design relies on the quality of documentation and may be subject to missing or misclassified clinical findings and screening timestamps. Second, this is a single-centre study, which may limit generalizability to settings with different antenatal detection pathways, staffing models, and discharge practices. Third, we did not conduct formal comparative analyses (e.g., pre- vs. post-implementation comparisons or risk-adjusted modelling), which limits causal inference regarding the independent impact of POCC on outcomes. Fourth, screening was performed in some cases (during the bed crisis period) at a median of ~12 hours of life, after which we strictly adhered to the 24-hour screening protocol, since earlier timing may influence false-positive rates compared with protocols recommending screening after 24 hours [1]. Finally, our dataset was designed to evaluate in-hospital detection and immediate screening performance; longer-term outcomes such as readmissions, late diagnoses after discharge, neurodevelopmental impact, and cost-effectiveness were not assessed.

## Conclusions

In conclusion, a layered screening approach combining antenatal ultrasound, structured clinical examination, and universal POCC supported early identification of CCHD prior to discharge in our setting. POCC contributed to the detection of clinically well newborns with potentially serious cardiac lesions that might otherwise have remained unrecognised at initial assessment. In addition, several screen-positive infants were found to have clinically significant non-cardiac hypoxemic conditions requiring further evaluation.

These findings highlight the complementary role of POCC alongside antenatal and clinical screening within a comprehensive detection pathway. Continued adherence to standardised screening protocols, along with ongoing audit and quality improvement efforts, may help optimise early detection strategies for CCHD within the neonatal care system.
